# Variation in Pollen-Donor Composition among Pollinators in an Entomophilous Tree Species, *Castanea crenata*, Revealed by Single-Pollen Genotyping

**DOI:** 10.1371/journal.pone.0120393

**Published:** 2015-03-20

**Authors:** Yoichi Hasegawa, Yoshihisa Suyama, Kenji Seiwa

**Affiliations:** 1 Graduate School of Agricultural Science, Tohoku University, Osaki, Miyagi, Japan; 2 Institute of Wood Technology, Akita Prefectural University, Noshiro, Akita, Japan; University of New South Wales, AUSTRALIA

## Abstract

**Background:**

In plants, reproductive success is largely determined by the composition of pollen (i.e., self-pollen and outcross-pollen from near and distant pollen-donors) transported as a result of pollinator foraging behavior (e.g., pollen carryover). However, little evidence is available on how and to what extent the pollen carryover affects the pollen-donor composition and on which insect taxa are effective outcross-pollen transporters under field conditions. In this study, we explored roles of foraging behavior of insect pollinators on pollen-donor composition and subsequent reproductive success in a woody plant.

**Methods:**

We performed paternity analyses based on microsatellite genotyping of individual pollen grains found on diurnal pollinators (i.e., bumblebee, small bee, fly, small beetle, and honeybee) visiting *Castanea crenata* trees.

**Results:**

The outcross-pollen rate was highest in bumblebees (66%), followed by small bees (35%), flies (31%), and small beetles (18%). The effective number of pollen donors, representing pollen carryover, was greater in bumblebees (9.71) than in flies (3.40), small bees (3.32), and small beetles (3.06). The high percentages of pollen from outside the plot on bumblebees (65.4%) and flies (71.2%) compared to small bees (35.3%) and small beetles (13.5%) demonstrated their longer pollen dispersal distances.

**Conclusions:**

All of the diurnal insects carried outcross-pollen grains for long distances via pollen carryover. This fact suggests that a wide range of insect taxa are potential outcross-pollen transporters for the self-incompatible *C*. *crenata*.

## Introduction

Pollination is a fascinating process in which immobile plants frequently use mobile animals to mate with spatially separated conspecifics. The pollination system is an important model for the co-evolution between plants and animals [1,2]. Specialized mutualistic interactions between plants and pollinators have long been recognized: Stebbins [3] hypothesized that a plant species should evolve to maximize visits by its most effective pollinator. However, recent studies have revealed a generalized pollination system in which a variety of insects visit a given plant species [4,5]. Aigner [6] demonstrated that such a generalized system can be an adaptive strategy for plants where higher fitness is achieved through multiple pollinator species than through a single one. However, little evidence is available about the relative efficiencies of multiple pollinators in increasing plant fitness, mainly owing to difficulties in observing the behavior of multiple pollinators in the field.

Pollinators of different taxa frequently have varying pollination efficiencies that can be measured by several factors (i.e., pollen removal from anthers, pollen deposition on stigmas, seed production) in a variety of plant species (e.g., [7–11]). Movement patterns of pollen grains also differ among insect pollinators, mainly because of their variable foraging behaviors [12–19]. For example, beetles of the subfamily Cetoniinae (*Protaetia cataphracta* and *Eucetonia pilifera*) carried greater amounts of outcross pollen much farther (maximum distance = 1100 m) than bumblebees (*Bombus ardens* and *B*. *diversus*) and small beetles (*Arthromacra sumptuosa*) in a low-density population of the hermaphrodite tree *Magnolia obovata* [16,17]. These facts suggest that pollen-dispersal patterns strongly affect plant fitness, particularly when closely related individual plants are distributed in a spatially aggregated manner (i.e., a fine-scale genetic structure) and potentially subject to biparental inbreeding depression [20–23].

Insect pollinators usually deposit very small fractions of the pollen grains they carry on each flower visited, with the remainder being carried to the next flowers and plants (i.e., pollen carryover; [24–30]). When pollen carryover occurs, the pollen grains attached to the surface of an insect’s body may include those derived from different individual plants (i.e., mixed paternity), increasing the rate of outcrossing rather than self-pollen grains being deposited on stigmas [25,26,28,30]. Long-distance dispersal of the outcross-pollen grains may also be facilitated by carryover from several donor plants [28,30]. Furthermore, the extent of pollen carryover and the subsequent pollen composition may vary among pollinator groups, probably because of variations in morphology, size, and foraging behaviors (e.g., grooming bumblebees, non-grooming small beetles). Thus, plant reproductive success should be evaluated in the context of visitations by multiple pollinator groups, but the pollen carryover has not been investigated for multiple pollinator groups under natural conditions because of difficulties involved in paternity analysis of pollen grains found on an insect.

In this study, we evaluated the pollen-donor composition (i.e., self-pollen and outcross-pollen from near and distant donors) on individual pollinators that visited a deciduous broadleaf tree species, *Castanea crenata*, by DNA amplification and paternity analysis based on microsatellite genotyping of individual pollen grains. DNA amplification of pollen grains or pollinaria (pollen packages) found on a pollinator has been reported in several recent studies using amplified fragment-length polymorphisms (AFLP; [31]), chloroplast DNA [32], a nuclear ribosomal internal transcribed spacer (ITS; [33–35], and nuclear microsatellites [16,17,36–39]. Multiplex polymerase chain reaction (PCR) techniques that amplify nuclear microsatellite regions in a single reaction allow for easy paternity analysis from a single pollen grain [16,17,38–41].

To assess the extent of pollen carryover, which can be represented by the pollen-donor diversity on a pollinator’s body, we evaluated the effective number of pollen donors (1/2F_S_; [42]) within a pollen pool found on a pollinator as the reciprocal of the mean of correlated paternity (2F_S_: proportion sharing the same donor tree between two pollen grains), which was calculated using 11 microsatellite markers. The effective number of pollen donors within a pollen pool has been frequently used to measure pollen-donor diversity (e.g., multiple paternity within a fruit; [30,43–45]) because sample size (i.e., the number of seeds or pollen grains; [43]) has little effect.

To understand the pollinator effectiveness of a wide range of insect taxa under field conditions, we performed a detailed investigation of the pollen-donor compositions of pollen pools found on individual pollinators visiting *C*. *crenata*. We specifically addressed the following questions: (1) Which groups of insects carry more outcross-pollen grains? (2) Does the amount of pollen carryover differ among insect groups? If so, (3) do the insect groups with greater pollen carryover transport pollen farther?

## Materials and Methods

### Plant material

*Castanea crenata* Sieb. et Zucc. (Japanese chestnut) is a deciduous broadleaved tree that is common in temperate forests of Japan [46]. The genus *Castanea* has two types of inflorescence: unisexual staminate catkins at proximal positions on the shoot and bisexual catkins at terminal positions [47]. Multiple female flowers are present at the bases of the bisexual catkins. Only the male flowers produce nectar [48], but the female flowers typically require pollinators for seed production [49]. Each female flower develops into a single cupule after pollination. Flowering occurs between late June and early August at the study site, with male and female flowering periods usually overlapping within individual plants. As a result, the self-pollination rate is very high (90.2%), whereas the selfing rate at the seed stage is very low, 0.3% [41], probably owing to late-acting self-incompatibility [50]. Pollen disperses an average of 43 m in *C*. *crenata*, with a maximum of 242 m observed in a natural forest [41].

### Study site

The study site was located in a deciduous broadleaved forest dominated by *Quercus serrata* and *C*. *crenata* at the Field Science Center, Tohoku University (38°45′N, 140°45′E), northeastern Japan. We mapped all of the trunks of *C*. *crenata* (*n* = 60; S1 Fig.) in a 1.6 ha (100 × 160 m) plot. *C*. *crenata* is single trunked, so that there were 60 individual trees in this plot. In 2005, all of the trees flowered during insect-collection period (13–19 July), so we considered them to be potential pollen donors. Leaf tissues for DNA microsatellite genotyping were collected from each of the 60 trees. The sample plot was located at the edge of a large *C*. *crenata* population that bordered conifer plantations on the northwest and southwest sides (S1 Fig.).

### Field sampling

We collected insects that visited *C*. *crenata* flowers using an insect net at the top of a tree tower from 13 to 19 July 2005. The tower enabled us to collect the insects visiting four adult trees (S1–S3 Figs.). Each insect collected was individually placed in a film case to prevent cross-contamination of pollen grains among pollinators. Because pollinators rarely visited the female flowers, we collected them from male flowers only. We collected 1975 pollen grains from 30 individual pollinators from the following five groups: bumblebee (*n* = 6), small bee (*n* = 5), fly (*n* = 8), small beetle (*n* = 9), and honey bee (*n* = 2; Table 1). These insects were collected during the daytime and stored at −30°C prior to DNA analysis. None of the nocturnal floral visitors (i.e., the small beetle *Nacerdes caudata*; S2d Fig.) carried any pollen grains, so we did not perform pollen DNA analysis for nocturnal insects.

**Table 1:** List of insect pollinators.

Pollinator group	ID	Species
Bumblebee	Bb1	*Bombus ardens*
	Bb2	*B*. *hypocrita* subsp. *hypocrita*
	Bb3	*B*. *hypocrita* subsp. *hypocrita*
	Bb4	*B*. *diversus* subsp. *diversus*
	Bb5	*B*. *ardens*
	Bb6	*B*. *diversus* subsp. *diversus*
Small bee	Sb1	*Lasioglossum ebmerianum*
	Sb2	*L*. *nipponicola*
	Sb3	*L*. *nipponicola*
	Sb4	*L*. *nipponicola*
	Sb5	*Andrena miyamotoi*
Fly	Fl1	*Mallota munda*
	Fl2	Tachinidae sp.
	Fl3	*Takanoa hakusana*
	Fl4	*Musca conducens*
	Fl5	*Kramerea schuetzei*
	Fl6	*Eristalis tenax*
	Fl7	*E*. *tenax*
	Fl8	*E*. *tenax*
Small beetle	Bt1	*Hoplia moerens*
	Bt2	*Hoplia moerens*
	Bt3	*Hoplia moerens*
	Bt4	*Hoplia moerens*
	Bt5	*Arthromacra viridissima*
	Bt6	*Harmonia axyridis*
	Bt7	*Hoplia moerens*
	Bt8	*Hoplia moerens*
	Bt9	*Hoplia moerens*
Honeybee	Hb1	*Apis cerana* subsp. *japonica*
	Hb2	*A*. *cerana* subsp. *japonica*

**Fig 1:**
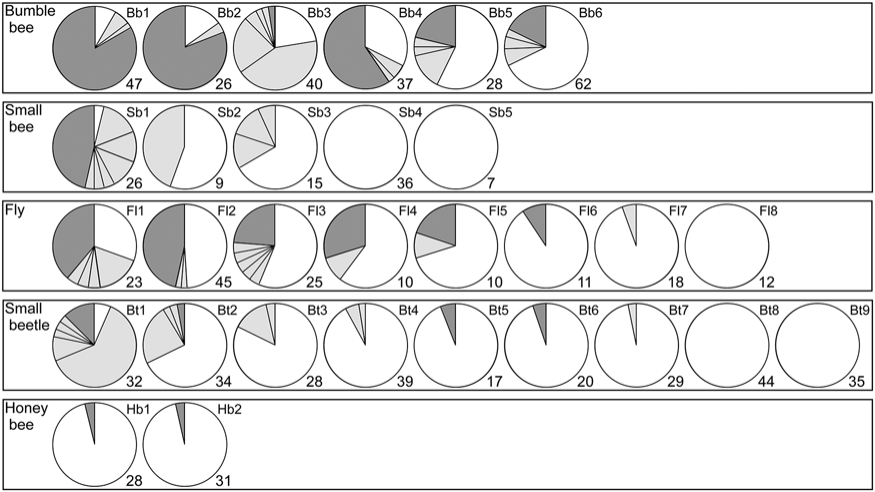
Pollen-donor compositions found on insects visiting *Castanea crenata*. Each pie chart represents an individual insect. White segments represent self-pollen, light segments individual pollen donors from within the plot, and dark segments pollen donors from outside the plot. Numbers of pollen grains tested for paternity are indicated. IDs for individual insects correspond to Table 1.

The pollen grains were collected from these insects for genotyping. IDs for individual insects correspond to those in Fig. 1.

### Treatment of pollen grains

Pollen grains were collected from the lower parts of the thorax and abdomen of insects using pipette tips. The pollen grains were placed in 0.01% sodium dodecyl sulfate (SDS) solution on slides with a water-repellent finish. Pollen grains that were morphologically identified as *C*. *crenata* and bore no structural damage were collected with a micropipette under a stereo dissecting microscope.

### DNA extraction and genotyping

Microsatellite genotypes of pollen grains were analyzed according to previous studies [40,41,51,52], with some modifications. One pollen grain and 0.5 μL of 0.01% SDS solution were placed in a PCR tube. After five rounds of freezing and thawing using −30°C ethanol and 50°C water, 1.0 μL of reaction buffer (10 mM Tris-HCl, pH 8.3 at 20°C; 1.5 mM MgCl_2_; 50 mM KCl; 0.01% Proteinase K) was added to the PCR tube, which was then incubated for 60 min at 54°C and heated for 10 min at 95°C. The extract was used directly as a PCR template. Total DNA from leaf tissues was isolated using a DNeasy 96 Plant Mini Kit (Qiagen, Hilden, Germany) according to the manufacturer’s protocol. PCR was performed in a GeneAmp PCR System 9600 (Applied Biosystems, Foster City, CA, USA). The forward primers were labeled with fluorescent dye (G5 dye set: 6-FAM, VIC, NED, or PET; Applied Biosystems) to simultaneously analyze 11 microsatellite loci of a similar allelic size and to avoid overlaps among loci with the same dye (Table 2). Furthermore, to avoid saturation of peak height on multiplex genotyping, we mixed fluorescent and non-fluorescent forward primers in appropriate ratios (Table 2). Multiplex PCR amplification was carried out using a Multiplex PCR Kit (Qiagen) in a 6.0-μL volume containing 1× Qiagen Multiplex PCR Master Mix, 0.2 μM of each primer, and 1.5 μL of template extract from a pollen grain or 0.5 μL of template from leaves. We used the following thermal cycler conditions: 94°C for 15 min (hotstart), 40 cycles at 94°C for 30 s, 57°C for 90 s, and 72°C for 1 min, followed by a final step at 60°C for 30 min. PCR products were electrophoresed on an ABI PRISM 3100-Avant Genetic Analyzer (Applied Biosystems), and allele fragment sizes were determined using GeneScan 3.0 and Genotyper 2.1 (Applied Biosystems). Pollen grains are haploid, but in some pollen samples, two alleles were found at one locus, indicating that two pollen grains had been placed in one PCR tube and then amplified at the same time. These samples were excluded from subsequent analyses, which were also restricted to pollen samples that had more than six genotyped loci.

**Table 2:** Characteristics of the microsatellite loci, including the type of fluorescent dye (Dye) and the mixing ratio of fluorescent and non-fluorescent forward primers (Fl: Non-fl), the observed number of alleles (*N*_A_), observed (*H*_O_) and expected (*H*_E_) heterozygosity, probability of exclusion when one parent is known (*P*_EX_), and estimated null allele frequency by locus for the 60 individual *Castanea crenata* trees at the Field Research Center, Tohoku University, Miyagi, Japan.

Locus	Dye	Fl : Non-fl	N_A_	H_O_	H_E_	P_EX_	Null allele frequency	Reference
CsCAT2	VIC	2 : 8	6	0.450	0.415	0.247	− 0.0571	Marinoni et al. [65]
CsCAT5	PET	10 : 0	13	0.717	0.681	0.459	+ 0.0334	Marinoni et al. [65]
CsCAT14	6-FAM	1 : 9	6	0.733	0.669	0.415	− 0.0502	Marinoni et al. [65]
EMCs2	PET	1 : 9	3	0.617	0.588	0.031	− 0.0223	Buck et al. [66]
EMCs17	PET	2 : 8	2	0.383	0.333	0.138	− 0.0750	Buck et al. [66]
KT001b	6-FAM	2 : 8	12	0.683	0.675	0.430	− 0.0061	Yamamoto et al. [67]
KT004a	VIC	4 : 6	12	0.750	0.790	0.594	+ 0.0254	Yamamoto et al. [67]
KT005a	NED	8 : 2	13	0.917	0.859	0.708	− 0.0369	Yamamoto et al. [67]
KT020a	NED	2 : 8	5	0.633	0.535	0.324	− 0.1258	Yamamoto et al. [67]
KT024a	6-FAM	1 : 9	4	0.317	0.296	0.157	− 0.0382	Yamamoto et al. [67]
KT030a	NED	3 : 7	7	0.867	0.757	0.537	− 0.0726	T. Yamamoto, pers. comm.
Mean			7.5	0.642	0.600	0.997*		

These parameters were calculated using CERVUS 3.0.3 [54].

*Overall combined exclusion probability

### Paternity analysis

Pollen samples that shared the same alleles at all analyzed loci as the tree from which the insects were collected were considered to be self-pollen. The paternity of each outcross-pollen grain was assigned by a simple exclusion approach based on the multilocus genotypes of the 60 trees. If a pollen grain did not match any potential pollen-donor genotypes among the 60 candidate trees, we assumed that it came from outside the study plot. If a pollen grain had two or more possible pollen-donor candidates, we inferred paternity based on a maximum-likelihood paternity assignment using CERVUS 3.0.3 [53,54]. The natural logarithm of the likelihood ratio of the loci was termed the logarithm of odds score (LOD) [55]. Marshall et al. [53] defined the Δ statistic as the difference in LOD scores between the most-likely and second most-likely male. CERVUS 3.0.3 performs a simulation to find critical values of Δ for strict and relaxed confidence levels (95% and 80% by default, respectively). The simulation parameters for CERVUS were as follows: 100,000 tests; the number (*n* = 60) of all individuals in the study plot; the proportion of candidate parents sampled (60%); the proportion of loci typed (90%); and a typing error rate of 0%. When the significance of the paternity analysis was less than 80%, or, when more than one individual shared the same LOD score, paternity was assigned to the spatially nearest individual with the highest positive LOD score.

### Outcross-pollen rate

For each insect, the outcross-pollen rate was determined directly by paternity assignment. The outcross-pollen rate was defined as the ratio of outcross-pollen grains to total pollen grains, including self-pollen grains. We compared the outcross-pollen rates among pollinator groups by applying a generalized linear mixed model (GLMM) with binomial errors and a logistic link using the glmmML package in R [56]. Here, individual insects were treated as random effects, and pollinator group (bumblebee, small bee, fly, or small beetle) as the fixed effect, with the pollen donor (outcross or self) as the response variable. We repeated this procedure for the six possible pairs of pollinator groups. We used the Bonferroni-corrected *P*-value (0.008) instead of 0.05. We excluded the honeybee from this and the following analyses because of the small sample size.

### Effective number of pollen donors

To assess pollen carryover, we applied a correlated paternity (proportion sharing the same donor tree between two pollen grains; [42]) using 11 microsatellite markers. Computations were carried out using SPAGeDi 1.4 [57] by encoding pollen-grain genotypes and designating them as homozygous. We used J. Nason’s multilocus kinship estimator, described by Loiselle et al. [58], to calculate the correlated paternity. The Steel–Dwass test was used to evaluate the differences in mean correlated paternity among pollinator groups. The reciprocal of the mean of correlated paternity (2F_S_) corresponds to the effective number of pollen donors (1/2F_S_).

### Pollen-dispersal distances

To compare the pollen-dispersal distances of outcross-pollen grains among insect groups, we distinguished between the outcross-pollen grains originating from inside and outside the study plot and then compared the frequencies of pollen dispersal from outside the study plot among insect groups using a chi-squared test followed by Ryan’s post hoc test. The pollen-dispersal patterns of insect-pollinated tree species shows a long-tailed distribution (e.g., [17,59,60]), and this dispersal pattern was observed in *C*. *crenata* [41]; thus, we considered pollen dispersal from outside the study plot as longer-distance dispersal than that from inside of the plot. All statistical analyses were performed using R 2.10.0 [56].

## Results

### DNA amplification from pollen grains

For 896 (45.4%) of the 1975 pollen samples isolated from insects, DNA fragments were successfully amplified and genotyped at more than six loci. In 72 (8.0%) of the 896 samples, we obtained two alleles at one locus, and these were excluded from our analysis, leaving microsatellite genotypes for a total of 824 pollen grains. The total numbers of pollen grains used for subsequent analyses per insect group (per individual insect) were: 240 (26–62), 93 (7–36), 154 (10–45), 278 (17–44) and 59 (28–31) for the bumblebee, small bee, fly, small beetle, and honeybee, respectively (Fig. 1).

### Outcross-pollen rate

**Fig 2:**
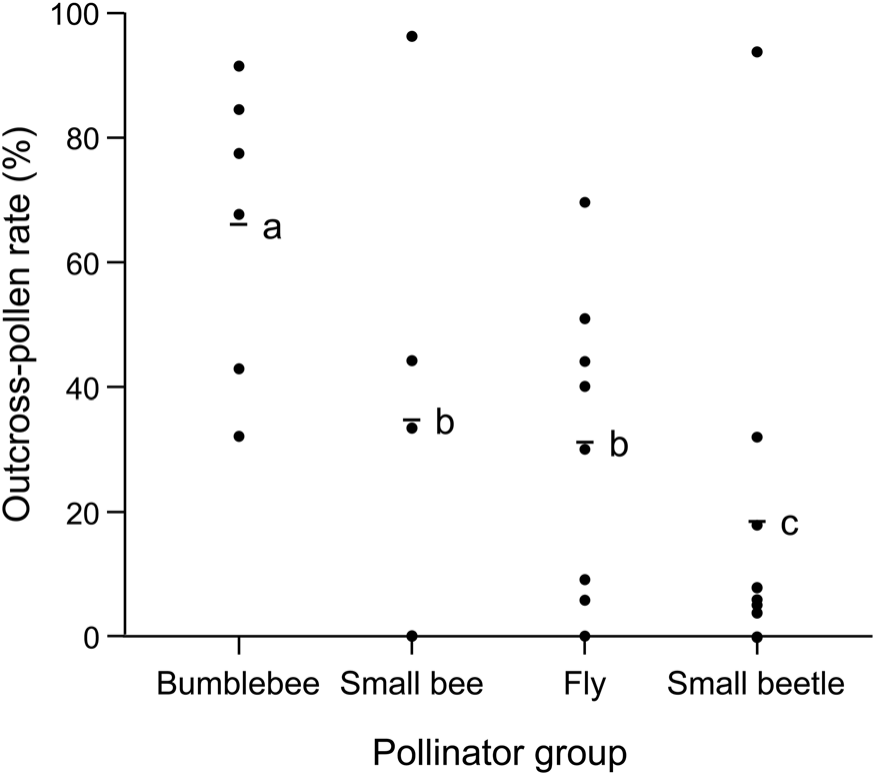
Proportion of outcross-pollen grains found on insects visiting *Castanea crenata*. Circles and bars represent each individual insect samples and the average value for each insect group, respectively. Values with different letters are significantly different at *P* < 0.008 (GLMM with Bonferroni adjustment). Bumblebee, *n* = 6; small bee, *n* = 5; fly, *n* = 8; small beetle, *n* = 9.

Of the pollen grains analyzed (*n* = 824), 300 (36.4%) were identified as outcross-pollen. The outcross-pollen rate was greatest in the bumblebee (mean ± SE; 66 ± 10%), followed by the small bee (35 ± 18%), fly (31 ± 9%), and small beetle (18 ± 10%; GLMM, *P* < 0.008; Fig. 2). The rate was not statistically different between the small bee and fly (GLMM, *P* > 0.008; Fig. 2).

### Effective number of pollen donors

**Fig 3:**
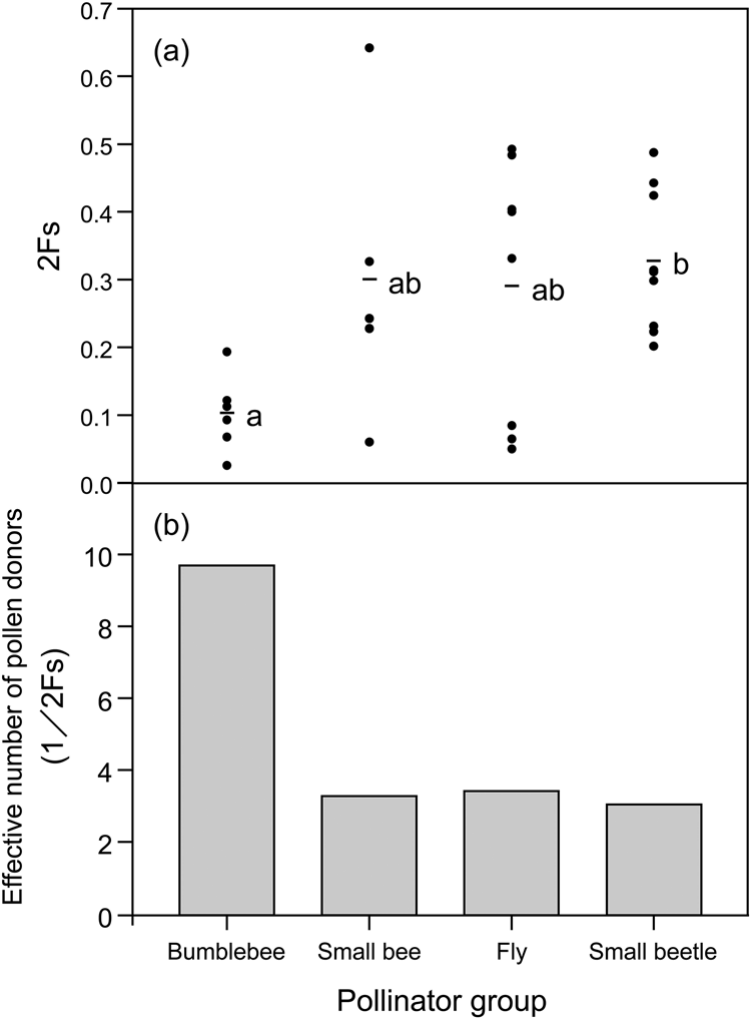
Correlated paternity and effective number of pollen donors. (a) Correlated paternity between two pollen grains within a pollen pool found on insects visiting *Castanea crenata*. Circles and bars represent individual insect samples and the average value for each insect group, respectively. Values with different letters are significantly different at *P* < 0.05 (Steel–Dwass test). (b) Effective number of pollen donors was the reciprocal of the mean of correlated paternity.

The correlated paternity between two pollen grains (2F_S_) for the bumblebee (mean ± SE; 0.103 ± 0.023) was lower than that for the small beetle (small beetle, 0.327 ± 0.034; Steel–Dwass test, *P* < 0.05; Fig. 3a), and there was little difference among the other insect groups (small bee, 0.301 ± 0.096; fly, 0.294 ± 0.069; Steel–Dwass test, *P* > 0.05; Fig. 3a). Greater variations in 2F_S_ were observed among individuals within pollinator group (Fig. 3a). An exception was the bumblebee, whose effective number of pollen donors (1/2F_S_: the reciprocal of the mean of correlated paternity) was significantly higher than that for the small beetle (9.71 vs. 3.06; Fig. 3b).

### Pollen dispersal distance

**Fig 4:**
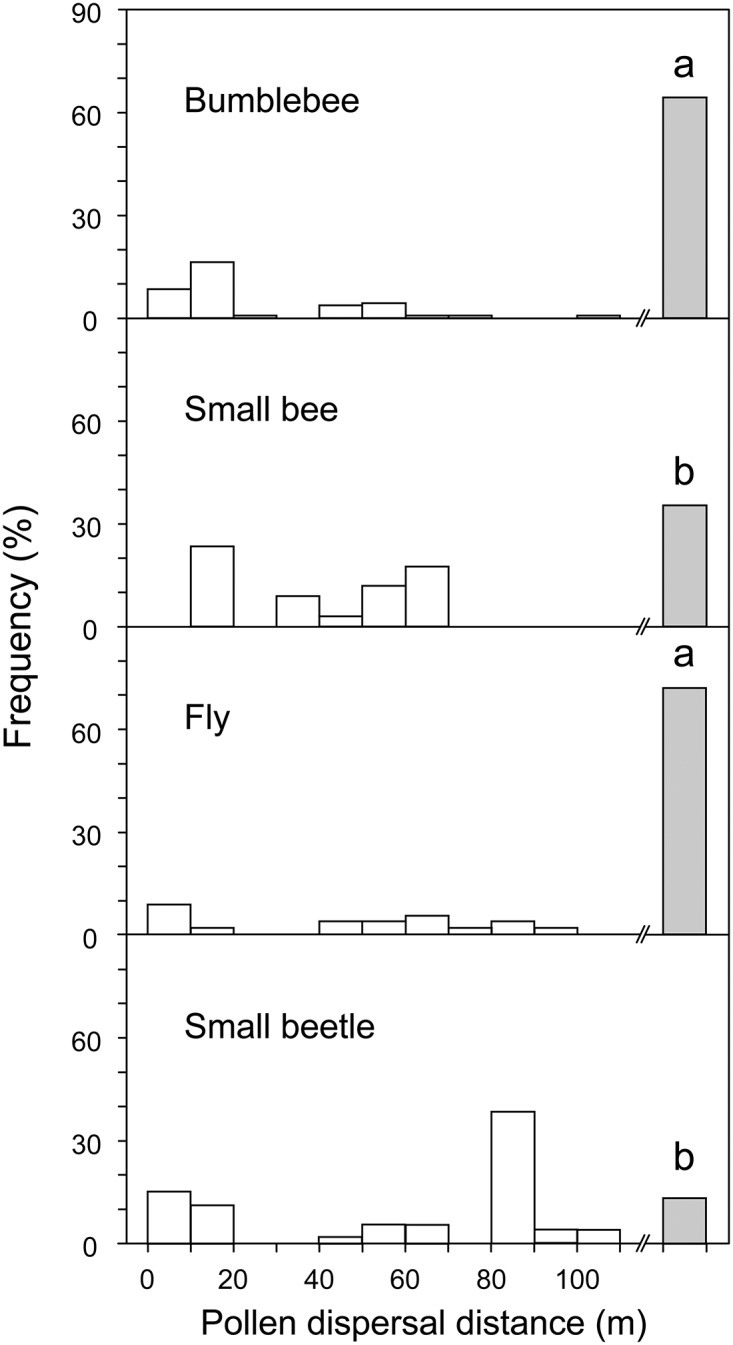
Histograms showing distances between assigned pollen donors and the trees from which insects were collected. Gray bars indicate the proportion of pollen from outside the plot found on insects. Values with different letters are significantly different at *P* < 0.05 (χ^2^ test followed by Ryan’s method as post hoc test).

For 163 (54.3%) of the 300 outcross-pollen grains, the donors were outside the plot area. Furthermore, the ratios of pollen from outside the plot were higher for the bumblebee (65.4%) and fly (71.2%) than for the small bee (35.3%) and small beetle (13.5%, χ^2^ test followed by Ryan’s method as a post hoc test, *P* < 0.05, Fig. 4). Thus, the dispersal distance of pollen grains was greatest for the bumblebee and fly.

## Discussion

Microsatellite genotyping of individual pollen grains clearly showed that the pollen-donor composition differed greatly among pollinators in a natural population of *Castanea crenata*. To our knowledge, this is the first study documenting the variations in outcross-pollen rates, effective number of pollen donors, and pollen dispersal distances among pollinators under field conditions. We also found that these variations could be attributed to the marked differences in the extent of pollen carryover among pollinators with variable foraging behaviors.

### Effective pollinators transporting outcross-pollen grains

In bumblebees, we found several pollen donors within pollen loads (i.e., pollen grains adhering to the insect; Fig. 1, S4 Fig.), suggesting that they frequently moved between *C*. *crenata* specimens, even though the individual trees had large flowering displays. As a result, bumblebees showed the greatest outcross-pollen rate (mean; 66%). To our knowledge, this is the first study showing that the bumblebee may be the most effective outcross-pollen transporter among pollinators visiting flowers of a hardwood tree species. In contrast, several previous studies have suggested that bumblebees frequently probe several flowers in sequence when they visit plants with large floral displays [61,62]. Recently Matsuki et al. [16] found that bumblebees collected pollen intensively from a single adult in a hermaphrodite tree, *Magnolia obovata*, resulting in a lower outcross-pollen rate (12%). Such differences in the foraging behavior of bumblebees may be attributed to the differences in tree density between *M*. *obovata* (1.2 ha^−1^; [16]) and *C*. *crenata* (38 ha^−1^ in this study site); the higher density population of *C*. *crenata* would result in lower foraging costs for bumblebees than that of *M*. *obovata*.

This study also suggests that the high effective number of pollen donors represented by bumblebees was largely caused by the high level of pollen carryover. Given that bumblebees usually carry larger pollen loads than other insect groups (e.g., small beetle; [16]), a greater proportion of outcross-pollen grains from several donor plants would remain on the body, even though replacement of outcross-pollen grains by self-pollen grains may have occurred frequently during the foraging sequence within a single tree. Furthermore, bumblebee grooming may uncover previously buried pollen and expose it to stigmas, thus enhancing pollen carryover and outcross-pollen deposition on the stigmas [63] (but see [29]). These traits agree with the prediction that more extensive pollen carryover would reduce geitonogamous self-pollination [25,26,28,30]. Harder & Wilson [27], however, pointed out that pollen-donor composition may differ among sites on a pollinator’s body that are groomed versus not groomed. Thus, we should test whether pollen-donor compositions differ among the sites of a bumblebee’s body by using single-pollen genotyping [64].

The effective number of pollen donors was lowest for the small beetle, indicating the lowest frequency of pollen carryover. This result probably occurred because most of the outcross-pollen grains were replaced by self-pollen grains on the body during the foraging sequence within a tree, resulting in a decreased outcross-pollen rate (18%).

In both the small bee and fly, the effective number of pollen donors (i.e., pollen carryover) and the outcross-pollen rates were intermediate between those of the bumblebee and small beetle and showed a positive relationship. The evidence suggests that pollen carryover has a positive effect on outcross-pollen deposition in the natural population, and an insect's efficiency as an outcross-pollen transporter varies widely among the insect groups.

### Pollen dispersal distance

Recent molecular studies have demonstrated that insect pollinators disperse pollen across long distances (e.g., 242–16000 m) in several tree species [17,41,59,60]. Pollen-dispersal distances strongly affect plant fitness, particularly when closely related individual plants are spatially aggregated (i.e., have a fine-scale genetic structure) because of biparental inbreeding depression [20–23]. Our paternity analyses based on microsatellite genotyping of individual pollen grains clarified the relative importance of insect groups in transporting pollen grains from outside the study plot, indicating long-distance pollen dispersal in *C*. *crenata*. Here, both the bumblebee and fly were more effective pollen transporters than the small bee and small beetle. We also revealed that this long-distance dispersal was achieved through pollen carryover, suggesting that long-distance pollen dispersals are mediated by the accumulation of several movements by the pollinators among individual trees (S4 Fig.). However, we have yet to understand whether the carryover mechanism increases the pollen dispersal distances of the bumblebee and fly in low-density *C*. *crenata* populations. Further experiments will be required to reveal the mechanisms of pollen carryover in tree populations of different densities and for a variety of pollinators.

### Conclusions and future research

A variety of diurnal insects (i.e., bumblebee, small bee, fly, and small beetle) carried outcross-pollen grains for a long distance through pollen carryover in a natural population of *C*. *crenata*, although the outcross-pollen rate varied among pollinator groups. Furthermore, we observed that the outcross-pollen rate varied within a pollinator group and even within a species (e.g., *Hoplia moerens*; small beetle). Thus, to determine the general trend of pollen-donor composition within a particular category or species of pollinators, further studies measuring much greater numbers of pollinators within each category or species are needed.

In *C*. *crenata*, only outcross-pollen grains are used for seed production because the trees are self-incompatible [41,50]. Insects from a wide range of taxa may act as potential pollinators for this species, although the bumblebee may be the most effective outcross-pollen transporter. In this study, we collected insects from male but not female flowers, because insects rarely visited the latter. Although we did not observe whether the insects actually transported pollen to female flowers, our study demonstrated the potential of outcrossing by insects. Further studies of the visiting frequencies of insects to female flowers and pollen deposition by insects will reveal the true effectiveness of these insects as pollinators of *C*. *crenata*.

## Supporting Information

S1 Fig Location of the four insect-capture trees (stars) and the other 56 trees (filled circles) of *Castanea crenata* in the plot. Gray areas indicate conifer plantations.
(PDF)

S2 Fig Flower-visiting insects. (a) Diurnal bumblebee, *Bombus ardens*, male, (b) fly, Oestroidea, and (c) small beetle, *Hoplia moerens* and (d) nocturnal small beetle, *Nacerdes caudata* feeding in flowers of insect-capture trees of *Castanea crenata*. Photographs by Miki Konno and Yoichi Hasegawa.
(PDF)

S3 Fig Canopy observation system (12 m tall). Photograph by Miki Konno.
(PDF)

S4 Fig Distribution of pollen-donor trees (filled letters), insect-capture trees (open circles), and other trees (dots), connected with their nearest neighboring pollen-donor trees based on pollen grains brought by an individual insect. (PDF)
