# Supplementary material for: Variation in Pollen-Donor Composition among Pollinators in an Entomophilous Tree Species, Castanea crenata, Revealed by Single-Pollen Genotyping
Source: PLoS One. 2015 Mar 20;10(3):e0120393. doi: 10.1371/journal.pone.0120393 (PMC4368697; doi:10.1371/journal.pone.0120393)
Supplement: S1 Fig — Gray areas indicate conifer plantations. (PDF) [file pone.0120393.s001.pdf]

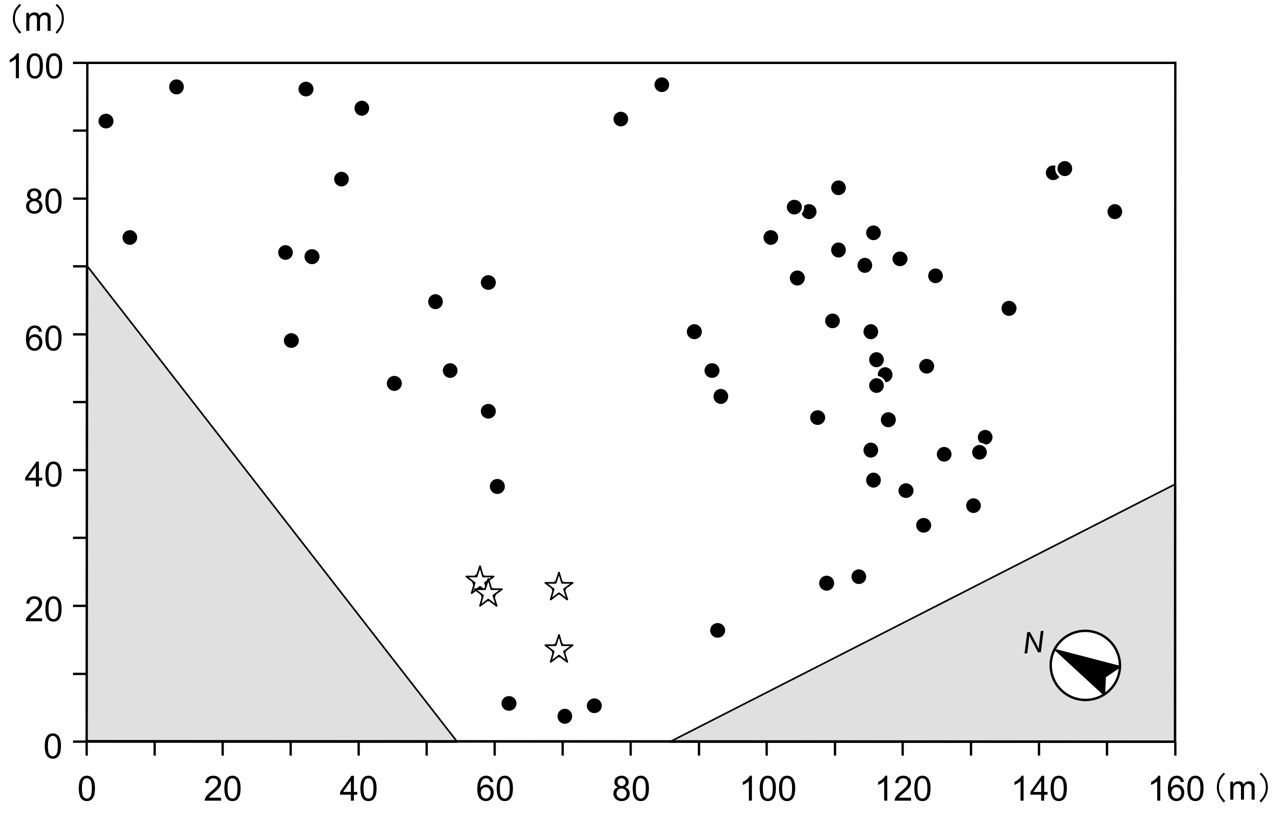

Figure S1 Location of the four insect-capture trees (stars) and the other 56 trees (filled circles) of *Castanea crenata* in the plot. Gray areas indicate conifer plantations.
