# Supplementary material for: Variation in Pollen-Donor Composition among Pollinators in an Entomophilous Tree Species, Castanea crenata, Revealed by Single-Pollen Genotyping
Source: PLoS One. 2015 Mar 20;10(3):e0120393. doi: 10.1371/journal.pone.0120393 (PMC4368697; doi:10.1371/journal.pone.0120393)
Supplement: S2 Fig — (a) Diurnal bumblebee, Bombus ardens, male, (b) fly, Oestroidea, and (c) small beetle, Hoplia moerens and (d) nocturnal small beetle, Nacerdes caudata feeding in flowers of insect-capture trees of Castanea crenata. Photographs by Miki Konno and Yoichi Hasegawa. (PDF) [file pone.0120393.s002.pdf]

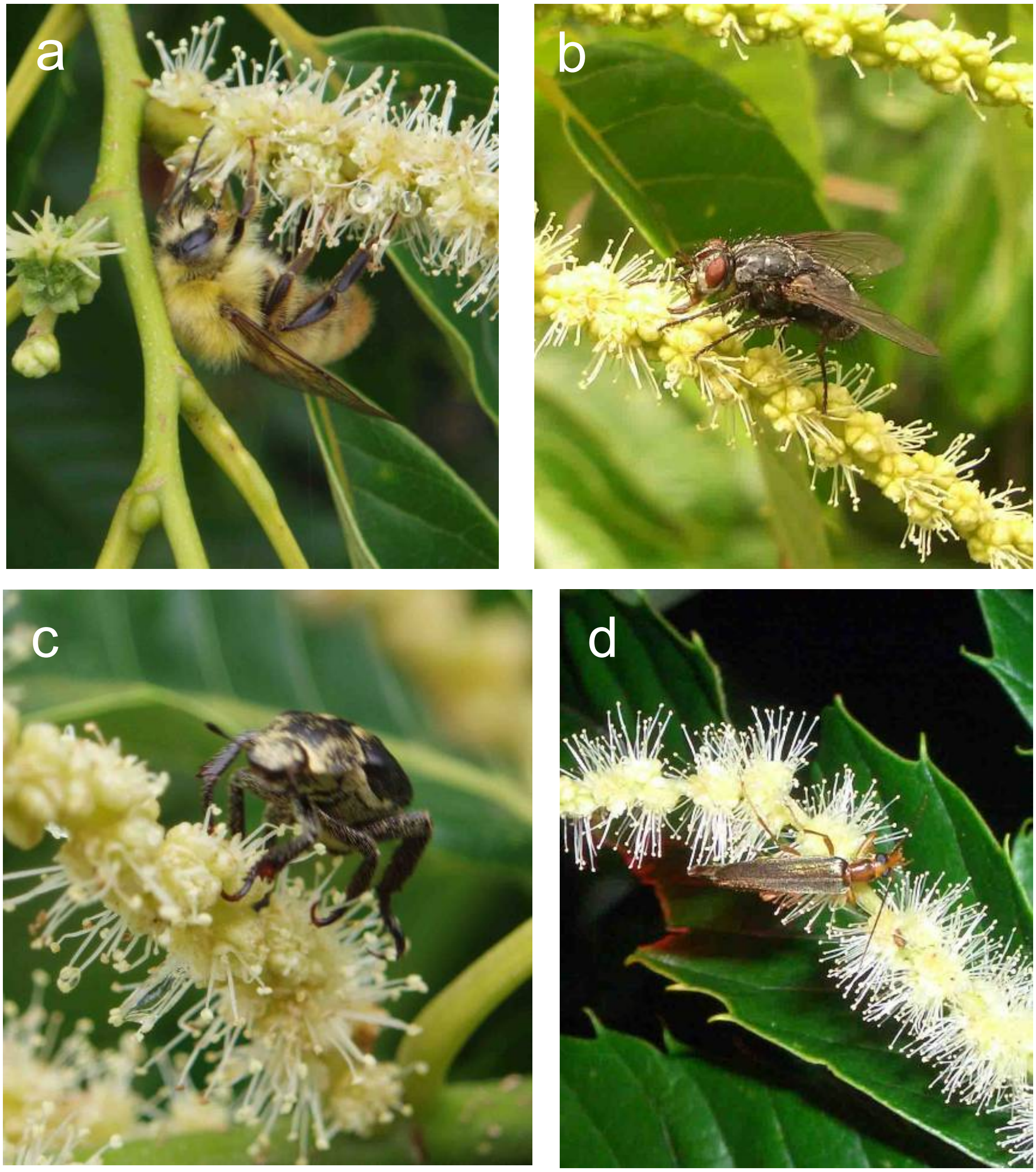

Figure S2 Flower-visiting insects. (a) Diurnal bumblebee, *Bombus ardens*, male, (b) fly, Oestroidea, and (c) small beetle, *Hoplia moerens* and (d) nocturnal small beetle, *Nacerdes caudata* feeding in flowers of insect-capture trees of *Castanea crenata*. Photographs by Miki Konno and Yoichi Hasegawa.
