# Supplementary figures and images for: Variation in Pollen-Donor Composition among Pollinators in an Entomophilous Tree Species, Castanea crenata, Revealed by Single-Pollen Genotyping
Source: PLoS One. 2015 Mar 20;10(3):e0120393. doi: 10.1371/journal.pone.0120393 (PMC4368697; doi:10.1371/journal.pone.0120393)

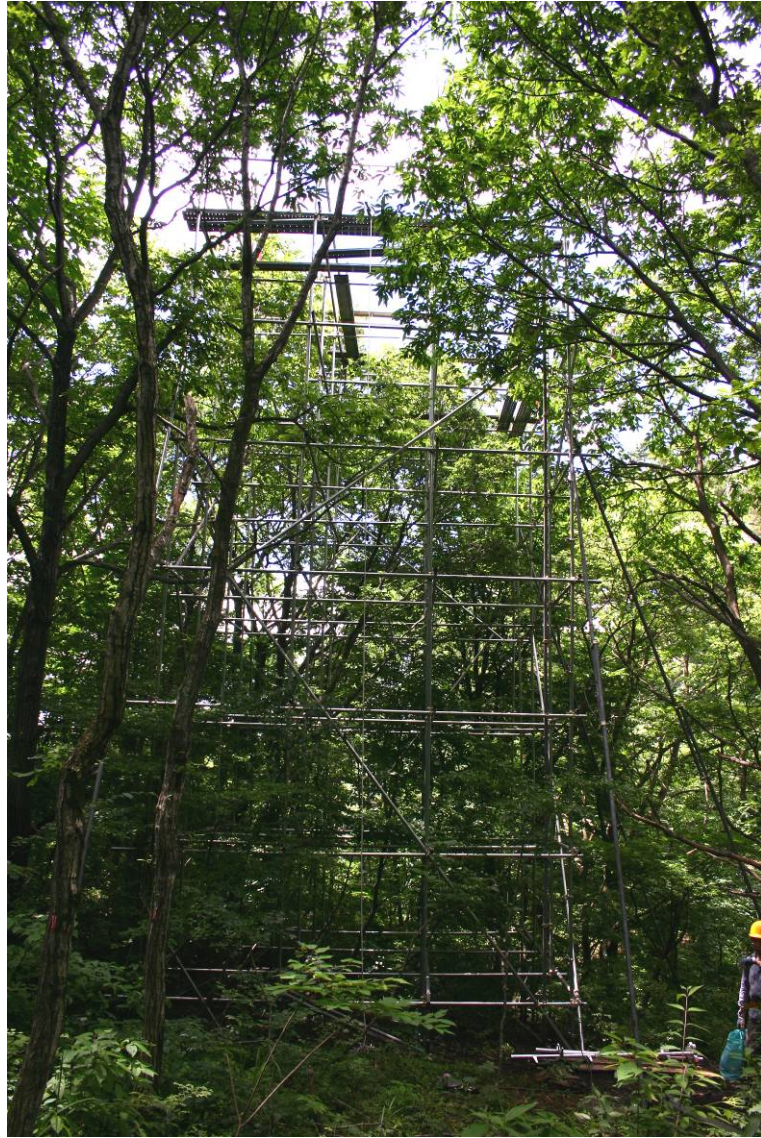

**Figure S3** Canopy observation system (12 m tall). Photograph by Miki Konno.

Supplement: S3 Fig — Photograph by Miki Konno. (PDF) [file pone.0120393.s003.pdf]
