# Supplementary material for: Variation in Pollen-Donor Composition among Pollinators in an Entomophilous Tree Species, Castanea crenata, Revealed by Single-Pollen Genotyping
Source: PLoS One. 2015 Mar 20;10(3):e0120393. doi: 10.1371/journal.pone.0120393 (PMC4368697; doi:10.1371/journal.pone.0120393)
Supplement: S4 Fig — (PDF) [file pone.0120393.s004.pdf]

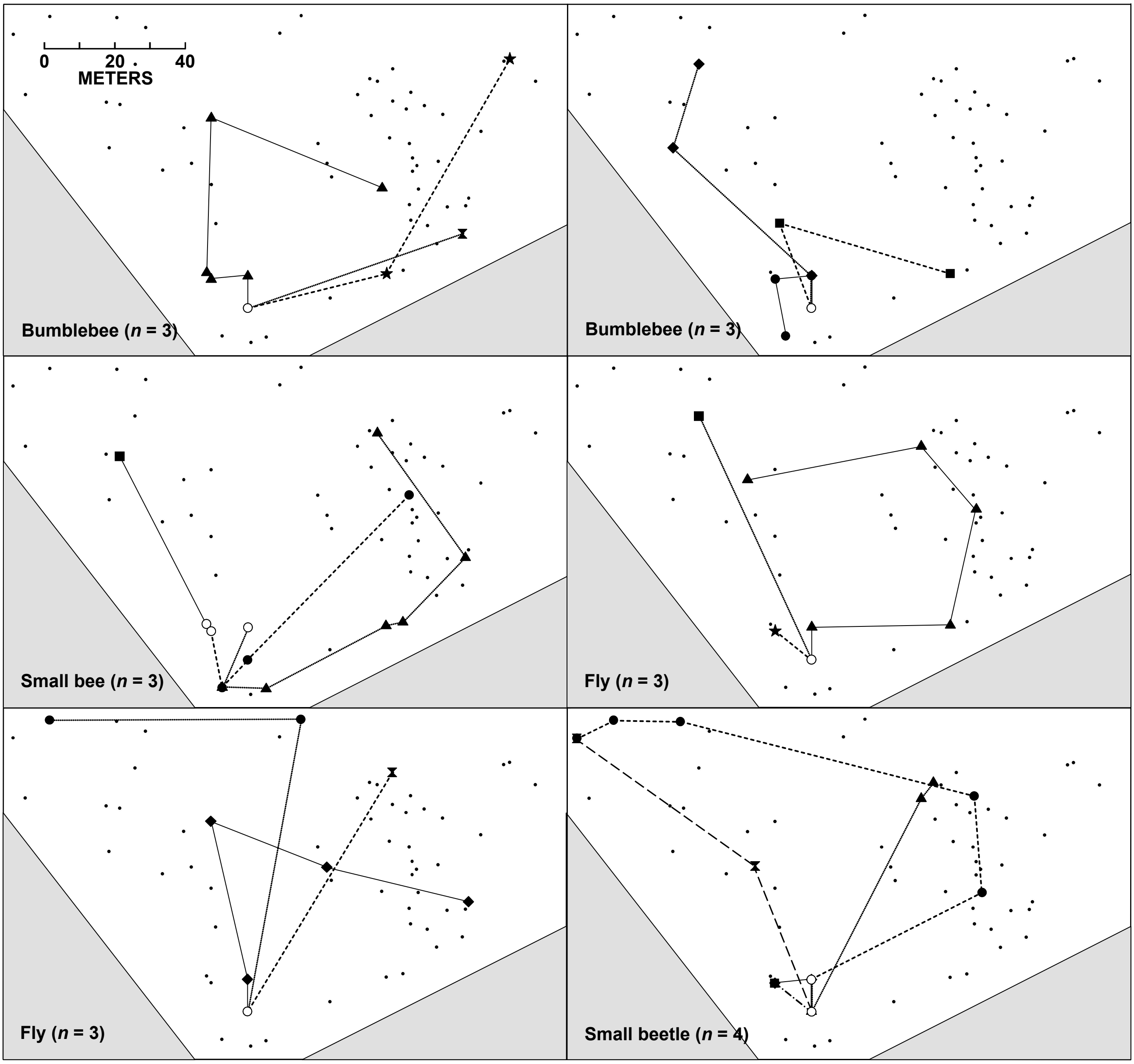

Figure S4 Distribution of pollen-donor trees (filled letters), insect-capture trees (open circles), and other trees (dots), connected with their nearest neighboring pollen-donor trees based on pollen grains brought by an individual insect.
